# 

**Published:** 1913-06

**Authors:** 


					﻿PUBLISHED MONTHLY.
SUBSCRII’TION $1.00
ATLANTA
JOURNAL-REG^gS
OF MEDICINE
(Atlanta Medical and Surgical Journal, Established 1555. ) CA|L Vanp
Suwessor to|||Ild southern Medical Record, Established 1870.	) OUlll YCdi
Vol. LX.
Atlanta, Ga., June, 1913.
No. 3
				

